# Monitoring of ADHD Medication: Are We in Line With NICE Guidelines? a Closed Loop Audit

**DOI:** 10.1192/bjo.2022.459

**Published:** 2022-06-20

**Authors:** Clare Langan

**Affiliations:** NHS Greater Glasgow and Clyde, Glasgow, United Kingdom

## Abstract

**Aims:**

Attention Deficit Hyperactivity Disorder (ADHD) in adults is a growing clinical problem and its prevalence among patients being referred to the General Adult Psychiatry clinic is rapidly increasing. The treatment of ADHD involves the use of medications such as methylphenidate, atomoxetine and lisdexamfetamine. These medications can cause significant adverse effects including arrhythmias, hypertension and appetite suppression. NICE guidelines stipulate that individuals on such medications should have weight, blood pressure and heart rate monitored every 6 months. The aim of this closed-loop audit was to assess if weight, heart rate and blood pressure are being monitored in line with current NICE guidelines in those who are on medication to treat ADHD in a Community Mental Health Team in Glasgow.

**Methods:**

Patients with an ADHD diagnosis were identified through a search of electronic case records. Electronic records were reviewed for each patient identified to assess if weight, heart rate and blood pressure had been recorded in the last 6 months. The results of the first cycle of this audit was presented at a local meeting in May 2021 with relevant clinicians present. The patient cohort identified was subsequently re-audited in December 2021 to assess if there had been an improvement in the monitoring of these medications.

**Results:**

30 patients were identified who had an ADHD diagnosis. 15 male and 15 female patients were identified. Patient age ranged from 18–50. 10 patients did not engage with services and were so subsequently excluded from our analyses. There was a substantial improvement in the monitoring of weight, heart rate and blood pressure in the second cycle compared with the first cycle of this audit. 45% of patients had their weight recorded (previously 15%), 40% had their heart rate recorded (previously 8%) and 50% had their blood pressure monitored (previously 19%).

**Conclusion:**

There has been a significant improvement in monitoring heart rate, blood pressure and weight every 6 months in line with NICE guidelines in the second cycle compared with the first cycle of this audit. However, we are still not currently meeting NICE guidelines. This is of particular clinical significance given the increasing prevalence of patients with an ADHD diagnosis and subsequent increase in the use of these medications. The COVID-19 pandemic and the reduction in face-to-face reviews has likely had an impact on our ability to monitor these medications.

